# Supplementary material for: The Possible Synergistic Pharmacological Effect of an Oral Berberine (BBR) and Curcumin (CUR) Complementary Therapy Alleviates Symptoms of Irritable Bowel Syndrome (IBS): Results from a Real-Life, Routine Clinical Practice Settings-Based Study
Source: Nutrients. 2024 Apr 18;16(8):1204. doi: 10.3390/nu16081204 (PMC11053504; doi:10.3390/nu16081204)
Supplement: Supplementary file 1 [file nutrients-16-01204-s001.zip › nutrients-2936931-supplementary.pdf]

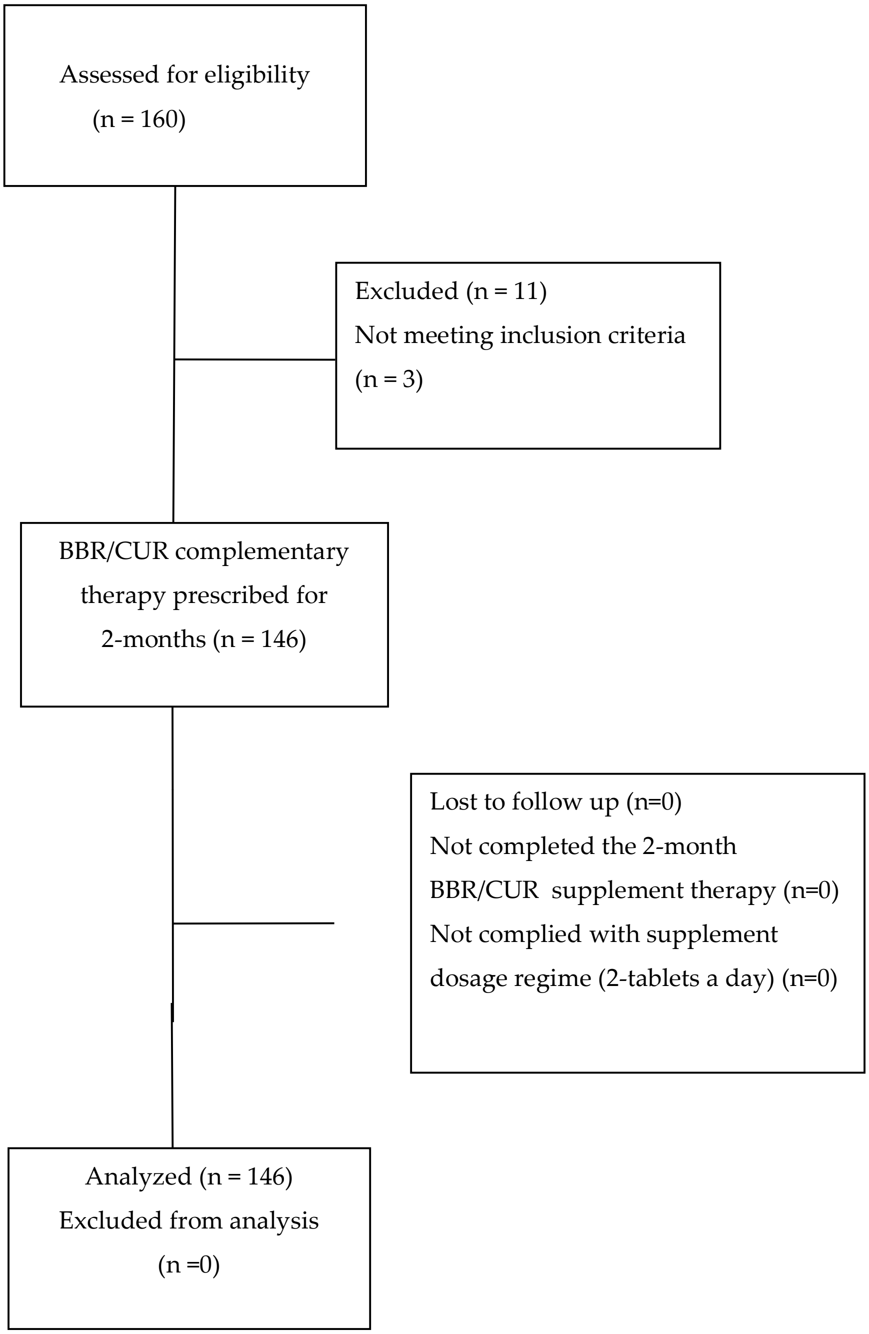

Figure S1. Study CONSORT flow diagram.

Table S1. Patient's population on concomitant IBS-D, IBS-C, and other drug treatments (N=146).

Subgroup	N (%)
Antidiarrheic alone (IBS-D)	8 (5.5)
Laxative alone (IBS-C)	17 (11.6)
Both antidiarrheic and laxative	6 (4.1)
Other drugs	115 (78.8)

Table S2. IBS-D patients symptoms severity (mean ± SD) at T_0 and T_1 (N=8).

Variable	N	T_0	T_1	Decrease between T_0 and T_1		
		Score	Score	Score	95%CI	P-value
**Patient's IBS symptoms**						
Abdominal pain	8	44.4 ± 26.1	24.8 ± 14	19.6 ± 19.6	3.3 - 36	0.025
Abdominal distension	8	53.8 ± 26.7	19.1 ± 14.3	34.6 ± 26.5	12.5 - 56.7	0.0076
Intestinal transit	8	65 ± 13.1	27.5 ± 7.6	37.5 ± 14.1	25.7 - 49.3	0.0001
Influence on quality of life	8	68.8 ± 14.6	25.6 ± 8.2	43.1 ± 20.2	26.3 - 60	0.0005
IBSSI	8	231.9 ± 57.3	97 ± 36.5	134.9 ± 52.8	90.7 - 179.1	0.0002
**Weekly Stool passage frequency**						
Minimum	8	13.1 ± 8.7	8.4 ± 5.2	4.8 ± 6.2	-0.4 to -9.9	0.067
Maximum	8	24.5 ± 14.5	14.9 ± 12.1	9.6 ± 5.2	5.3 to -14.0	0.0012

T_0: Baseline visit, T_1: After 2-months BBR-CUR supplementation follow-up visit.

Table S3. IBS-C patients symptoms severity (mean ± SD) at T_0 and T_1 (N=17)

Variable	N	T_0	T_1	Decrease between T_0 and T_1		
		Score	Score	Score	95%CI	P-value
**Patient's IBS symptoms**						
Abdominal pain	17	64.1 ± 13	40.3 ± 15.7	23.8 ± 14.5	16.4 - 31.3	<.0001
Abdominal distension	17	63.5 ± 16.2	43.5 ± 17.6	20 ± 18.1	10.7 - 29.3	0.0003
Intestinal transit	17	69.7 ± 14.8	47.6 ± 19.2	22.1 ± 25.1	9.2 - 34.9	0.0023
Influence on quality of life	17	62.4 ± 12.5	44.4 ± 17.1	17.9 ± 14.3	10.6 - 25.3	<.0001
IBSSI	17	259.7 ± 39.2	175.9 ± 60.6	83.8 ± 62.5	51.7 - 116	<.0001
**Weekly Stool passage frequency**						
Minimum	16	2.6 ± 2.2	5.2 ± 3.8	-2.6 ± 3.5	-4.4 to -0.7	0.0097
Maximum	17	9.3 ± 8.5	8.8 ± 5.8	0.5 ± 6.8	-3.0 to -4.0	0.78

T_0: Baseline visit, T_1: After 2-months BBR-CUR supplementation follow-up visit.

STROBE Statement—Checklist

	Item No	
**Title and abstract**	1	(*a*) Indicate the study's design with a commonly used term in the title or the abstract✓
		(*b*) Provide in the abstract an informative and balanced summary of what was done and what was found✓
**Introduction**		
Background/rationale	2	Explain the scientific background and rationale for the investigation being reported✓
Objectives	3	State specific objectives, including any prespecified hypotheses✓
**Methods**		
Study design	4	Present key elements of study design early in the paper✓
Setting	5	Describe the setting, locations, and relevant dates, including periods of recruitment, exposure, follow-up, and data collection✓
Participants	6	Give the eligibility criteria, and the sources and methods of selection of participants. Describe methods of follow-up✓
Variables	7	Clearly define all outcomes, exposures, predictors, potential confounders, and effect modifiers. Give diagnostic criteria, if applicable✓
Data sources/ measurement	8	For each variable of interest, give sources of data and details of methods of assessment (measurement). Describe comparability of assessment methods if there is more than one group✓
Bias	9	Describe any efforts to address potential sources of bias. The primary outcome measure IBSSI was adjusted for baseline covariates. To reduce information and interpretation bias, the objective of the BBR-CBR supplement complementary treatment was clearly explained to each patient at inclusion visit. GPs and pharmacists enrolled patients sequentially as they arrived. There was no selection on any ground.✓
Study size	10	As a retrospective study, no formal sample size calculation was made but investigators expected to include at least N=100 patients in the study. A power calculation showed that with such a sample size and assuming a baseline IBSSI of 300 and a standard deviation (SD) of 80 points, a drop of 15% in IBSSI after treatment could be detected with at least 99% power at the 5% critical level. ✓
Quantitative variables	11	Explain how quantitative variables were handled in the analyses. If applicable, describe which groupings were chosen and why.✓
Statistical methods	12	(*a*) Describe all statistical methods, including those used to control for confounding✓
		(*b*) Describe any methods used to examine subgroups and interactions✓
		(*c*) Explain how missing data were addressed. Missing data were neither replaced nor imputed ✓
		(*d*) If applicable, explain how loss to follow-up was addressed✓
		(*e*) Describe any sensitivity analyses. Not done
**Results**		
Participants	13	(a) Report numbers of individuals at each stage of study—eg numbers potentially eligible, examined for eligibility, confirmed eligible, included in the study, completing follow-up, and analysed✓
		(b) Give reasons for non-participation at each stage✓

		(c) Consider use of a flow diagram✓
Descriptive data	14	(a) Give characteristics of study participants (e.g., demographic, clinical, social) and information on exposures and potential confounders✓
		(b) Indicate number of participants with missing data for each variable of interest✓
		(c) Summarise follow-up time (eg, average and total amount)✓
Outcome data	15	Report numbers of outcome events or summary measures over time✓
Main results	16	(*a*) Give unadjusted estimates and, if applicable, confounder-adjusted estimates and their precision ((e.g., 95% confidence interval). Make clear which confounders were adjusted for and why they were included✓
		(*b*) Report category boundaries when continuous variables were categorized✓
		(*c*) If relevant, consider translating estimates of relative risk into absolute risk for a meaningful time period
Other analyses	17	Report other analyses done—(e.g., analyses of subgroups and interactions, and sensitivity analyses. NA
**Discussion**		
Key results	18	Summarise key results with reference to study objectives✓
Limitations	19	Discuss limitations of the study, taking into account sources of potential bias or imprecision. Discuss both direction and magnitude of any potential bias✓
Interpretation	20	Give a cautious overall interpretation of results considering objectives, limitations, multiplicity of analyses, results from similar studies, and other relevant evidence✓
Generalisability	21	Discuss the generalisability (external validity) of the study results✓
**Other information**		
Funding	22	Give the source of funding and the role of the funders for the present study and, if applicable, for the original study on which the present article is based✓
